# Polymorphisms of the DNA Methyltransferase 1 Gene Predict Survival of Gastric Cancer Patients Receiving Tumorectomy

**DOI:** 10.1155/2016/8578064

**Published:** 2016-03-21

**Authors:** Zhifang Jia, Xing Wu, Donghui Cao, Chuan Wang, Lili You, Meishan Jin, Simin Wen, Xueyuan Cao, Jing Jiang

**Affiliations:** ^1^Division of Clinical Epidemiology, First Hospital of Jilin University, Changchun 130021, China; ^2^Division of Pathology, First Hospital of Jilin University, Changchun 130021, China; ^3^Department of Gastric and Colorectal Surgery, First Hospital of Jilin University, Changchun 130021, China

## Abstract

DNA methyltransferase 1 (DNMT1) plays a pivotal role in maintaining DNA methylation status. Polymorphisms of* DNMT1* may modify the role of DNMT1 in prognosis of gastric cancer (GC). Our aim was to test whether polymorphisms of* DNMT1* gene were associated with overall survival of GC. Four hundred and forty-seven GC patients who underwent radical tumorectomy were enrolled in the study. Five tagging SNPs (rs10420321, rs16999593, rs2228612, rs2228611, and rs2288349) of the* DNMT1* gene were genotyped by TaqMan assays. Kaplan-Meier survival plots and Cox proportional hazard regression were used to analyze the associations between SNPs of* DNMT1* and survival of GC. Patients carrying rs2228611 GA/AA genotype tended to live longer than those bearing the GG genotype (HR 0.68, 95% CI: 0.51–0.91, *P* = 0.007). Further multivariate Cox regression analysis showed that rs2228611 was an independent prognostic factor (GA/AA versus GG: OR 0.67, 95% CI 0.49–0.91, *P* = 0.010). Nevertheless, other SNPs did not show any significant associations with survival of GC. Polymorphisms of the* DNMT1* gene may affect overall survival of GC. The SNP rs2228611 has the potentiality to serve as an independent prognostic marker for GC patients.

## 1. Introduction

Gastric cancer (GC) is the most common gastrointestinal malignancy and the third leading cause of cancer-related death worldwide [1]. Although radical gastrectomy has been regarded as an effective treatment, prognosis for GC patients remains poor, with a 5-year overall survival (OS) rate of only 27.4% in China [2]. GC patients have different outcomes even when they are diagnosed at the same clinical stage and receiving the same treatment [3], which suggests that the host characteristics, specifically the genetic factors, play crucial roles in GC prognosis. Single nucleotide polymorphisms (SNPs) are the most common genetic variations and reportedly influence the patients' clinical outcomes [4–6].

DNA methylation, which is established and maintained by DNA methyltransferases (DNMTs), is the major direct modification of eukaryotic DNA and is known to have profound effects on the regulation of gene expression [7–10]. DNMT1 is the primary enzyme that maintains the level of DNA methylation [11]. Aberrant expression of DNMT1 is correlated with progression and prognosis of various cancers, such as lung cancer [12], hepatocellular carcinoma [13], pancreatic cancer [14], and bladder cancer [15]. In GC, overexpression of DNMT1 correlates with worse differentiation, advanced stage, and increased risk of death [16–18]. Meanwhile, inhibition or blockade of DNMT1 can arrest cell cycle, increase cell apoptosis, decrease invasion, and enhance treatment sensitivity [15, 16, 19].

Previous studies have reported that SNPs of* DNMT1* gene are associated with susceptibility to cancers such as ovarian cancer [20] and breast cancer [21]. In GC, though Khatami et al. found no association in an Iranian population [22], Yang et al. observed a 45% increased risk in individuals bearing rs16999593 C allele in Southern Chinese population [23]. Our previous study showed that rs10420321 GG or rs8111085 CC genotype was associated with lower risk of* Helicobacter pylori* infection but higher risk of gastric atrophy [24].

Given the role of DNMT1 in cancer progression and prognosis, we hypothesized that SNPs of* DNMT1* may have prognostic value of cancer. As far as we know, there was no study on it. The aim of our study was to explore the role of SNPs of* DNMT1* in the prognosis of gastric cancer [23].

## 2. Material and Methods

### 2.1. Subject Selection

Subjects of the study were described elsewhere [24, 25]. From August 2008 to December 2010, a total of 447 GC patients who underwent tumorectomy in the Department of Gastric and Colorectal Surgery of the First Hospital of Jilin University were recruited in this study. All of them were histologically diagnosed with gastric cancer and none of them received chemotherapy or radiotherapy before surgery. All patients were Han descent from the area of Changchun. Clinical and pathological data were collected including age, gender, tumor size, pathological type, differentiation, depth of invasion, lymph metastasis, distant metastasis, lymph-vascular invasion, and therapy. The clinical stage of gastric cancer was evaluated by the TNM classification according to American Joint Committee on Cancer (AJCC) in 2010, the seventh edition [26]. Written informed consent was obtained from each patient before sample collection. This study was approved by the ethics committee of the First Hospital of Jilin University.

The follow-up of patients was performed by telephone calls three months, six months, and one year after the tumorectomy and every one year thereafter until death or being lost to follow-up. The following data were recorded during each follow-up: the health status of patients, therapy after operation, and date of death if the patients died. About one-third of the patients received chemotherapy after surgery. The chemotherapy mainly consisted of three regimens: FOLFOX-4 regimen (combination with 5-fluorouracil, leucovorin, and oxaliplatin); XELOX regimen (capecitabine and oxaliplatin); and other chemotherapies such as capecitabine or 5-fluorouracil alone. A patient would be considered to have postoperative chemotherapy only if they received the therapy for at least 3 cycles.

### 2.2. Selection of Tagging SNPs

The detailed description of tagging SNPs selection was depicted in our previous work [24]. Briefly, tagging SNPs were selected from the Chinese Han data (CHB) in the HapMap Project (06-02-2009 HapMap) using the SNP browser Software v4.0 to capture SNPs with minimum minor allele frequency (MAF) > 0.05 and pairwise *r* ^2^ ≥ 0.8 [27]. There were 27 SNPs with MAF > 0.05 in* DNMT1* gene region in the Chinese on HapMap. Three of them lie in the coding regions, including two nonsynonymous SNPs of rs16999593 (His97Arg) in exon 4 and rs2228612 (Ile327Val) in exon 12 and one synonymous SNP rs2228611 in exon 17. The remaining 24 SNPs reside in introns of* DNMT1*. Five linkage disequilibrium (LD) blocks were identified. The 3 SNPs in coding region could tag the blocks they belonged to (*D*′ = 1 and *r* ^2^ = 0.8) and were selected. Finally, five SNPs, rs10420321, rs16999593, rs2228612, rs2228611, and rs2288349, were selected as tagging SNPs for further study.

### 2.3. Genotyping

Blood samples of each subject were collected in EDTA tubes and stored in −80°C until DNA extraction. Genomic DNA was extracted using commercial kits following the protocol provided by the manufacturer (AxyPrep Blood Genomic DNA Miniprep kit, Axygen Biosciences, USA). Genotypes of each SNP were determined by TaqMan SNP genotyping assays in 384-well plates (Applied Biosystems, USA) and the detailed process of polymerase chain reaction (PCR) was described elsewhere [24]. The PCR products were read on ABI PRISM 7900 Sequence Detector in the end-point mode and genotypes were identified using the Allelic Discrimination Sequence Detector Software V2.3. Ten randomly selected samples were genotyped twice in all SNPs to verify genotyping and sample-handling accuracy and the concordance rate was 100%. The call rates for rs10420321, rs16999593, rs2228612, rs2228611, and rs2288349 were 91.7%, 100%, 100%, 99.5%, and 99.3%, respectively.

### 2.4. Statistical Analysis

Overall survival was defined as the time from the date of surgery to the date of death (if the patients were dead) or to the date of final follow-up (if the patients were alive) or to the date of the last follow-up (if the patients were lost to follow-up). Survival time would be right-censored in case that the patients were alive, were lost to follow-up, or died of causes other than GC. Patients would be excluded from survival analysis if they were lost to follow-up at the first interview or they died of postoperative complications within 30 days such as uncontrollable bleeding during perioperative period.

Survival curves of the GC patients within each SNP were plotted by Kaplan-Meier method and compared by log-rank test. The median survival time was estimated by the Kaplan-Meier method. If median OS could not be calculated, mean OS would be used instead. Hazard ratios (HRs) with their 95% confidence intervals (CIs) were calculated by the Cox proportional hazards model after adjusting for age (scale variable), sex (nominal variable), clinical stage (scale variable), and postoperational chemotherapy (nominal variable). Stepwise Cox regression analysis was conducted to determine independent predictive factors of prognosis, with a significance level of *P* < 0.05 for entering and *P* > 0.05 for removing the variables. All analyses were performed using SPSS18.0 Software (SPSS Inc., USA). Two-tailed *P* values < 0.05 were considered to be statistically significant.

## 3. Results

### 3.1. Characteristics of Patients

A total of 447 patients with histologically confirmed GC were enrolled in the study. Twelve patients (2.7%) could not be connected any more after they left the hospital and follow-up information was available for 435 patients (97.3%) until March 2016. Thirteen patients (2.9%) died of postoperative complications. These 25 cases (12 + 13 = 25, 5.6%) were excluded from the analysis of effects of SNPs on survival. The remaining 422 patients were enrolled in further analysis. During the follow-up period, 197 (46.2%) patients died of gastric cancer, 19 (3.3%) died of other causes such as cerebral hemorrhage, 17 (2.4%) were lost to follow-up, and 189 (48.1%) were alive. The duration of follow-up ranged from 1.1 to 85.0 months with a median of 59.9 months (95% CI: 59.4–60.5 months).

The baseline characteristics and clinical features of these patients are detailed in Table 1. Of these patients, 72.3% were male gender, 87.4% were tubular adenoma type, 62.9% were poorly differentiated, 43.1% were at advanced stage (TNM III or IV), 96.4% received radical gastrectomy, and 32.7% received postoperational chemotherapy. Survival analysis shows that patients in male gender, with tumor size > 5 cm, with deeper invasion, with presence of lymph node metastasis or distant metastasis, with advanced TNM stage, positive for lymph-vascular or neural invasion, and receiving palliative surgery only had shorter postoperational survival compared to patients without (log-rank *P* < 0.05, Table 1).

### 3.2. Association between Genotypes and Survival of Gastric Cancer

As reported by our previous work [24], no association was found between the SNPs and pathological type, tumor differentiation, TNM stage, and lymph-vascular or neural invasion (Table 2).

**Figure 1:**
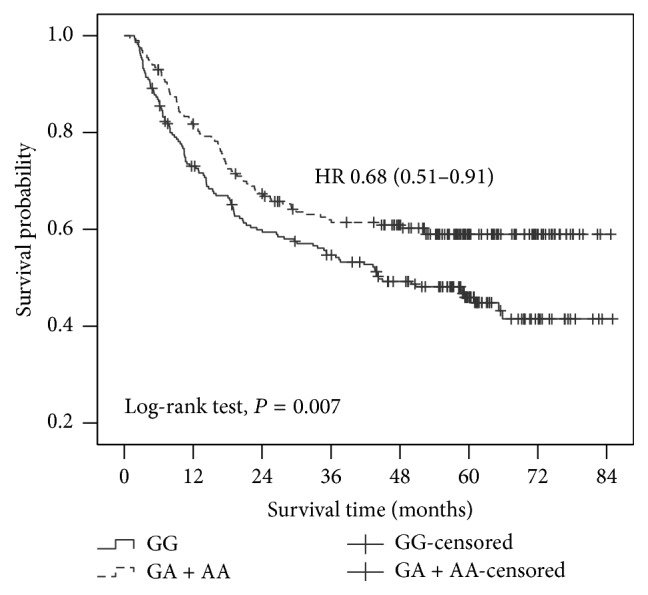
Association of* DNMT1* rs2228611 with overall survival in gastric cancer patients.

Survival curves within each SNP of* DNMT1* were plotted and compared using the codominant, dominant, and recessive model (Table 3). More than half of patients bearing the GG genotype of rs2228611 (117/221 = 52.9%) died of GC until the final follow-up, whereas only 39.7% of patients with the GA or AA genotype died (*P* = 0.007). Comparing to patients carrying the GG genotype of rs2228611, patients with GA or AA genotype had reduced risk of death after surgery (GA: HR 0.66, 95% CI 0.48–0.89; AA: HR 0.81, 95% CI 0.46–1.44). Result from the dominant model combining patients with GA or AA genotype showed that the A allele bearers of rs2228611 had 32% lower risk of death comparing to the GG carriers (HR 0.68, 95% CI 0.51–0.90, log-rank *P* = 0.007, Figure 1). Further subgroup analysis in patients receiving radical gastrectomy showed similar correlation where A allele reduced the death risk by 35% (HR 0.65, 95% CI 0.48–0.88, log-rank *P* = 0.005; adjusting for age, sex, TNM stage, and chemotherapy).

Other four SNPs, rs10420321, rs16999593, rs2228612, and rs2288349, were not associated with the overall survival of GC (Table 3). Further haplotype analysis did not show associations between the haplotype and survival in GC (Table 3).

### 3.3. Stratified Analysis between Genotypes and Survival

**Figure 2:**
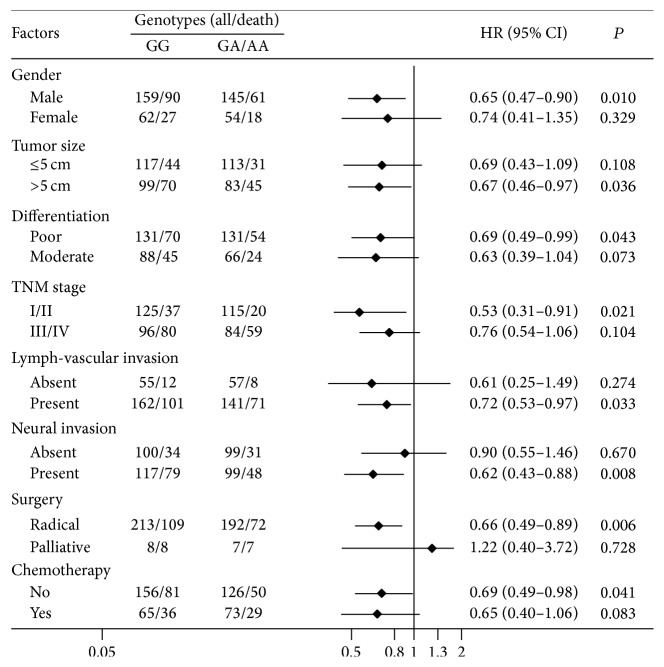
Stratified survival analysis for rs2228611 using the dominant model.

We further performed the stratified analysis for rs2228611 to investigate if this SNP on survival was modified by some important clinicopathological factors. As presented in Figure 2, patients carrying GA/AA variant genotypes had a lower death risk than those with the GG genotype. The protective effect was more pronounced among patients with relatively early clinical stage (HR 0.53, 95% CI: 0.31–0.91; *P* = 0.021).

### 3.4. Stepwise Cox Regression Model for GC Survival

Further multivariate Cox regression analysis showed that rs2228611 was an independent predictive factor for GC (GA/AA versus GG, HR 0.67, 95% CI 0.49–0.91, *P* = 0.010). Other factors, male gender (HR 1.80, 95% CI 1.22–2.67; *P* = 0.003), advanced TNM stage (HR 4.76, 95% CI: 3.28–6.90; *P* < 0.001), tumor size ≥ 5 cm (HR 1.56, 95% CI: 1.13–2.16; *P* = 0.007), and lymph-vascular invasion (HR 1.52, 95% CI: 1.08–2.14; *P* = 0.015), were associated with shorter survival of GC. In addition, receiving palliative surgery increased risk (HR 2.55, 95% CI: 1.40–4.64; *P* = 0.002) while receiving postsurgery chemotherapy (HR 0.69, 95% CI: 0.50–0.96; *P* = 0.029) decreased risk of death (Table 4).

## 4. Discussion

In the present study, we explored the associations of five SNPs of* DNMT1* gene (rs16999593, rs10420321, rs2288349, rs2228611, and rs2228612) with postoperational survival in Chinese gastric cancer patients. We found that rs2228611 was an independent prognostic factor where A allele of rs2228611 was associated with a 33% decreased risk of death (HR 0.67, 95% CI 0.49–0.91, *P* = 0.010, Table 4).

To the best of our knowledge, this is the first study to evaluate the prognosis value of* DNMT1* SNPs on cancer. Previous studies mainly focused on the role in tumorigenesis and they reported controversial results. Khatami et al. first assessed the associations of SNPs of* DNMT1* with sporadic colorectal cancer in 208 cases and 213 controls in Iranian population and they observed no significant correlation [28]. Liu et al. expanded the sample size (1609 colon cancer cases and 1974 controls) and found that rs2228612 together with dietary factors (folate, methionine, vitamin B2, and vitamin B12 intake) could modify risk of colon cancer [29]. Similar controversial results were reported in breast cancer [21, 30–32]. In gastric cancer, Khatami et al. did not find any association between SNPs of* DNMT1* (rs2228611, rs721186, rs11488, and rs13784) and disease risk in Iranian patients [22]. Yang et al. examined this association in the Southern Chinese population and found that the C allele of rs16999593 increased the GC risk by 45% (OR 1.45, 95% CI: 1.00–2.11, *P* = 0.05). Our previous study, however, found that rs2228612 CC genotype increased the risk of precancerous disease (gastric atrophy) but not GC [24]. In the present study, we assessed their roles in the prognosis of GC and observed that A allele of rs2228611 was associated with favorable postoperational survival (HR 0.67, *P* = 0.010).

Methylation of CpG islands in promoter region can lead to transcriptional silence of genes. Silencing genes related to tumor suppressors or invasion suppressors promote tumorigenesis and metastasis [33–35]. DNMT1 is a key enzyme to maintain DNA methylation pattern [11]. Upregulation of DNMT1 downexpresses genes such as P53, P21 via increasing methylation, which could enhance proliferation of cancer cells [36]. Inhibition of DNMT1 restores expression of tumor suppression-related genes such as p16 and RGS10 and contributes to reduced cancer cell viability, decreased invasive capability, and enhanced treatment sensitivity [15, 16, 19, 37]. Aberrant expression of DNMT1 is associated with unfavorable prognosis of various cancers including GC [16]. Evaluating the prognostic value of genetic variations in* DNMT1* in the prognosis of cancer makes sense. In our study, we found that the minor allele A of rs2228611 was associated with longer life-span of postoperational GC patients.

Rs2228611 is located in exon 17 of* DNMT1* and the G-to-A change mediates a synonymous variation (CCG→CCA, Proline→Proline). Bioinformatics tool SNPinfo predicts that rs2228611 is in the region of exonic splicing enhancer (ESE) [38]. The G-to-A variation may alter the binding activity to serine/arginine-rich (SR) protein, change pre-RNA splicing of* DNMT1*, and therefore lead to alteration of normal DNMT1 expression. However, the exact mechanism of rs2228611 contributing to the prognosis of GC is still unknown and needs further investigation.

Two limitations should be noted in our study. The first one was that we did not clarify the underlying specific function of rs2228611 in the prognosis of GC due to our study design. The second one was that the follow-up time was relatively short as 50.5% of the patients are alive and the right side of the survival plot was censored, although the median follow-up time was 54.5 months. Therefore, more studies with larger-scale and longer follow-up time are needed to confirm our finding and to clarify the exact mechanism.

## 5. Conclusions

In summary, our study provides the first evidence that polymorphisms of the* DNMT1* gene could modify the postoperational survival of gastric cancer cases. The A allele of rs2228611 contributes to favorable prognosis and has potentiality to serve as a prognostic predicting biomarker for gastric cancer.

## Figures and Tables

**Table 1:** Characteristics and clinical features of gastric cancer patients.

Variables	Number of patients	Number of deaths	Mean OS (months)	Log-rank *P*	HR (95% CI)
All	422	195	54.5		
Gender					
Female	117 (27.7)	45	57.8	0.044	1.00
Male	305 (72.3)	152	48.8		**1.41 (1.01–1.96)**
Age					
≤60	209 (49.5)	91	52.5	0.218	1.00
>60	213 (50.5)	106	50.2		1.19 (0.90–1.58)
Tumor size					
≤5 cm	231 (55.8)	75	62.6	<0.001	1.00
>5 cm	183 (44.2)	116	39.5		**2.63 (1.96–3.52)**
Pathological type					
Tubular adenocarcinoma	368 (87.4)	170	52.2	0.167	1.00
Signet ring cell carcinoma	22 (5.2)	8	49.1		0.75 (0.37–1.53)
Others	31 (7.4)	19	38.8		1.49 (0.93–2.40)
Differentiation					
Well to moderate	155 (37.1)	70	55.7	0.161	1.00
Poor	263 (62.9)	124	49.4		1.23 (0.92–1.65)
Depth of invasion					
T1/T2	97 (23.0)	13	74.2	<0.001	1.00
T3/T4	322 (77.0)	182	44.8		**5.96 (3.39–10.46)**
Lymph metastasis					
N0	119 (29.1)	15	75.5	<0.001	1.00
N1/N2/N3	300 (70.9)	180	42.0		**7.16 (4.22–12.14)**
Distant metastasis					
M0	392 (92.9)	174	53.9	<0.001	1.00
M1	30 (7.1)	23	25.4		**2.70 (1.75–4.18)**
TNM stage					
I/II	240 (56.9)	57	69.5	<0.001	1.00
III/IV	182 (43.1)	140	28.6		**5.97 (4.37–8.16)**
Lymph-vascular invasion					
Absent	112 (26.5)	20	73.4	<0.001	1.00
Present	305 (73.5)	173	43.9		**4.39 (2.76–6.99)**
Neural invasion					
Absent	199 (47.2)	65	63.0	<0.001	1.00
Present	218 (52.8)	128	42.4		**2.42 (1.80–3.27)**
Radical surgery					
Yes	407 (96.4)	182	53.6	<0.001	1.00
No^a^	15 (3.6)	15	10.8		**5.61 (3.28–9.60)**
Chemotherapy^b^					
None	284 (67.3)	132	51.7	0.157	1.00
XELOX	29 (6.9)	9	62.8		0.55 (0.28–1.08)
FOLFOX-4	77 (18.2)	36	51.3		0.98 (0.68–1.41)
Others	32 (7.6)	20	39.6		1.34 (0.84–2.15)

OS: overall survival; HR: hazard ratio; CI: confidence interval.

^a^Fifteen patients received palliative surgery.

^b^XELOX (capecitabine and oxaliplatin); FOLFOX-4 (5-fluorouracil, leucovorin, and oxaliplatin); other chemotherapies included 5-fluorouracil; Xeloda alone; paclitaxel plus leucovorin and tegafurum; LV5-FU2 (leucovorin plus 5-fluorouracil); and FOLFIRI (irinotecan, 5-fluorouracil, and leucovorin).

**Table 2:** Associations between the SNPs and clinical parameters of gastric cancer patients.

	rs10420321		*P*	rs16999593		*P*	rs2228612		*P*	rs2228611		*P*	rs2288349		*P*
	AA	AG + GG		TT	TC + CC		TT	TC + CC		GG	GA + AA		GG	GA + AA	
*N*	130	257		268	154		137	285		221	199		237	182	
Gender															
Male	31.9	68.1	0.334	62.4	37.6	0.532	31.4	68.6	0.650	53.3	46.7	0.885	55.2	44.8	0.538
Female	36.9	63.1		65.6	34.4		33.6	66.4		54.0	46.0		58.4	41.6	
Age															
≤60	34.5	65.5	0.600	64.7	35.3	0.571	34.0	66.0	0.392	54.0	46.0	0.837	52.6	47.4	0.154
>60	32.0	68.0		62.1	37.9		30.2	69.8		53.0	47.0		59.3	40.7	
Tumor size															
≤5 cm	33.6	66.4	0.982	64.8	35.2	0.668	33.1	66.9	0.807	50.6	49.4	0.400	57.0	43.0	0.933
>5 cm	33.5	66.5		62.8	37.2		31.9	68.1		54.7	45.3		56.6	43.4	
Pathological type															
Tubular adenocarcinoma	32.2	67.8	0.393	62.8	37.2	0.889	31.3	68.7	0.414	54.6	45.4	0.507	55.6	44.4	0.853
Signet ring cell carcinoma	36.8	63.2		65.2	34.8		30.4	69.6		47.8	52.2		56.5	43.5	
Others	43.8	56.3		66.7	33.3		42.4	57.6		45.5	54.5		60.6	39.4	
Differentiation															
Well to moderate	37.6	62.4	0.191	61.1	38.9	0.501	34.4	65.6	0.458	57.7	42.3	0.152	54.5	45.5	0.646
Poor	31.1	68.9		64.4	35.6		30.9	69.1		50.5	49.5		56.8	43.2	
TNM stage															
I/II	29.6	70.4	0.065	62.9	37.1	0.710	29.4	70.6	0.183	52.0	48.0	0.618	58.5	41.5	0.354
III/IV	38.4	61.6		64.6	35.4		35.4	64.6		54.4	45.6		54.1	45.9	
Lymph-vascular invasion															
Absent	33.0	67.0	0.849	60.7	39.3	0.499	31.6	68.4	0.800	49.6	50.4	0.446	57.8	42.2	0.829
Present	34.0	66.0		64.2	35.8		32.9	67.1		53.7	46.3		56.6	43.4	
Neural invasion															
Absent	32.8	67.2	0.699	62.6	37.4	0.794	33.0	67.0	0.848	50.0	50.0	0.305	60.0	40.0	0.215
Present	34.7	65.3		63.8	36.2		32.1	67.9		55.0	45.0		54.1	45.9	
Radical surgery															
Yes	33.2	66.8	0.995	63.7	36.3	0.415	31.9	68.1	0.910	53.5	46.5	0.991	56.2	43.8	0.827
No	33.3	66.7		53.3	46.7		33.3	66.7		53.3	46.7		53.3	46.7	
Chemotherapy															
No	30.2	69.8	0.056	59.9	40.1	0.028	28.7	71.3	0.026	56.4	43.6	0.069	55.1	44.9	0.530
Yes	39.8	60.2		70.7	29.3		39.3	60.7		47.1	52.9		58.3	41.7	

**Table 3:** Associations between the SNPs and overall survival of gastric cancer patients.

Genotypes	Overall patients *N* = 422		Mean OS	HR (95% CI)	*P*	Adjusted HR (95% CI)^a^	Adjusted *P*
	*n* (%)	Deaths					
rs10420321							
AA	130 (33.6)	62	50.0	1.00		1.00	
AG	189 (48.8)	85	53.6	0.90 (0.65–1.24)	0.510	0.91 (0.65–1.26)	0.553
GG	68 (17.6)	32	52.1	0.97 (0.63–1.49)	0.890	0.98 (0.64–1.50)	0.917
Dominant							
AA	130 (33.6)	62	50.0	1.00		1.00	
AG/GG	257 (66.4)	117	53.3	0.90 (0.67–1.24)	0.490	0.92 (0.68–1.26)	0.618
Recessive							
AA/AG	319 (82.4)	147	52.4	1.00		1.00	
GG	68 (17.6)	32	52.1	1.04 (0.71–1.52)	0.730	1.04 (0.70–1.52)	0.857
rs16999593							
TT	268 (63.5)	127	51.3	1.00		1.00	
TC	135 (32.0)	63	52.3	0.98 (0.72–1.32)	0.891	0.99 (0.73–1.34)	0.986
CC	19 (4.5)	7	50.5	0.80 (0.37–1.70)	0.555	0.80 (0.37–1.72)	0.802
Dominant							
TT	268 (63.5)	127	51.3	1.00		1.00	
TC/CC	154 (36.5)	70	52.9	0.96 (0.72–1.28)	0.767	0.96 (0.72–1.29)	0.801
Recessive							
TT/TC	403 (95.5)	190	51.8	1.00		1.00	
CC	19 (4.5)	7	50.5	0.80 (0.38–1.70)	0.471	0.81 (0.38–1.72)	0.576
rs2228612							
TT	137 (32.4)	65	50.1	1.00		1.00	
TC	197 (46.7)	90	52.9	0.92 (0.67–1.27)	0.629	0.92 (0.67–1.27)	0.614
CC	88 (20.9)	42	51.3	1.00 (0.68–1.47)	1.000	1.03 (0.70–1.53)	0.864
Dominant							
TT	137 (32.4)	65	50.1	1.00		1.00	
TC/CC	285 (67.6)	132	52.5	0.95 (0.70–1.27)	0.720	0.96 (0.71–1.29)	0.760
Recessive							
TT/TC	334 (79.1)	155	52.0	1.00		1.00	
CC	88 (20.9)	42	51.3	1.05 (0.75–1.47)	0.790	1.09 (0.77–1.53)	0.638
rs2228611							
GG	221 (52.6)	117	47.8	1.00		1.00	
GA	169 (40.3)	66	57.5	**0.66 (0.48–0.89)**	**0.006**	**0.65 (0.48–0.88)**	**0.006**
AA	30 (7.1)	13	45.9	0.81 (0.46–1.44)	0.469	0.80 (0.45–1.42)	0.444
Dominant							
GG	221 (52.6)	117	47.8	1.00		1.00	
GA/AA	199 (47.4)	79	56.7	**0.68 (0.51–0.90)**	**0.007**	**0.67 (0.51–0.90)**	**0.007**
Recessive							
GG/GA	390 (92.9)	183	52.1	1.00		1.00	
AA	30 (7.1)	13	45.9	0.96 (0.55–1.69)	0.894	0.95 (0.54–1.68)	0.860
rs2288349							
GG	237 (56.6)	102	54.7	1.00		1.00	
GA	151 (36.0)	77	47.4	1.27 (0.95–1.71)	0.1105	1.26 (0.94–1.70)	0.128
AA	31 (7.4)	17	46.0	1.39 (0.84–2.33)	0.204	1.37 (0.82–2.30)	0.228
Dominant							
GG	237 (56.6)	102	54.7	1.00		1.00	
GA/AA	182 (43.4)	94	47.7	1.29 (0.98–1.71)	0.072	1.28 (0.97–1.70)	0.086
Recessive							
GG/GA	388 (92.6)	179	52.4	1.00		1.00	
AA	31 (7.4)	17	46.0	1.27 (0.77–2.08)	0.353	1.25 (0.76–2.06)	0.376

OS: overall survival; HR: hazard ratio; CI: confidence interval.

^a^Adjusted for age, sex, TNM stage, and postoperational chemotherapy.

**Table 4:** Multivariate Cox regression analysis on the survival of GC.

Variables	HR	95% CI	*P*
rs2228611 (GA/AA versus GG)	0.67	0.49–0.91	0.010
Sex (male versus female)	1.80	1.22–2.67	0.003
TNM stages (III + IV versus I + II)	4.76	3.28–6.90	<0.001
Tumor sizes (≥5 cm versus <5 cm)	1.56	1.13–2.16	0.007
Lymph-vascular invasion (present versus absent)	1.52	1.08–2.14	0.015
Surgical method (palliative versus radical surgery)	2.55	1.40–4.64	0.002
Chemotherapy (yes versus no)	0.69	0.50–0.96	0.029

HR: hazard ratio; CI: confidence interval.
